# New-Onset Type 1 Diabetes in Children With SARS-CoV-2 Infection

**DOI:** 10.7759/cureus.22790

**Published:** 2022-03-03

**Authors:** Shashikanth Ambati, Maya Mihic, David Charles Rosario, Javier Sanchez, Adnan Bakar

**Affiliations:** 1 Division of Pediatric Critical Care, Albany Medical Center, Albany, USA; 2 Department of Pediatrics, Albany Medical Center, Albany, USA

**Keywords:** type 1 diabetes mellitus, pediatric endocrinology, pediatric intensive care unit (picu), diabetic ketoacidosis (dka), covid-19

## Abstract

There has been a worldwide increase in cases of diabetic ketoacidosis in both adults and children with diabetes during the severe acute respiratory syndrome coronavirus 2 (SARS-CoV-2) pandemic. This can be multifactorial: delayed care due to reduced medical services, fear of approaching hospitals, or SARS-CoV-2 infection itself. It is well-known that infection is an important trigger for diabetic ketoacidosis in children with type 1 or type 2 diabetes mellitus, but little is known whether SARS-CoV-2 infection can trigger diabetic ketoacidosis and new-onset diabetes mellitus in a child with no previous history of diabetes mellitus. The association of SARS-CoV-2 as a trigger for new-onset diabetes requires further investigation, as the incidence of diabetes is steadily rising in the pediatric population during the pandemic. This case report explores two cases where children present in diabetic ketoacidosis with concurrent SARS-CoV-2 infection and no known history of type 1 diabetes mellitus.

## Introduction

Adults with diabetes mellitus (DM) may be at higher risk of developing severe acute respiratory syndrome coronavirus 2 (SARS-CoV-2) with an increased risk of morbidity and mortality [1]. There have been reports worldwide on new-onset DM in SARS-CoV-2 patients and increased incidence of diabetic ketoacidosis (DKA) in those with pre-existing DM [2-4]. This trend has also been noted in children during the pandemic, with a rise in both the incidence of new-onset type 1 DM and presentation in DKA [5-8].

The question is whether SARS-CoV-2 infection is directly related to the increased incidence of DM and DKA in children or if the increased incidence is due to delay in seeking medical care due to the pandemic. Multiple hypotheses exist explaining how SARS-CoV-2 may trigger DM or DKA including SARS-CoV-2 directly binding to angiotensin-converting enzyme-2 (ACE-2) receptors in the pancreas resulting in islet cell damage or SARS-CoV-2 antibodies triggering an inflammatory process leading to beta-cell destruction and onset of DM [9-10].

Here, we present two cases of children presenting with new-onset DM in DKA and incidentally found to be SARS-CoV-2 positive. Though there are some case reports from other countries, there are rare cases reported in the United States. This case series of overt DKA associated with SARS-CoV-2 infection in two previously healthy children are being reported in view of its rarity.

## Case presentation

Case 1

A 10-year-old female presented with a five-day history of nausea, vomiting, diarrhea, and altered mental status. Her initial laboratory testing was consistent with DKA (Table 1). Her urinalysis was 4+ positive for ketones. She was also found to be SARS-CoV-2 positive by polymerase chain reaction (PCR). SARS-CoV-2 antibodies were not evaluated. Her mother endorsed her own positivity for SARS-CoV-2 infection two weeks prior to her child’s presentation. The patient was given appropriate fluid resuscitation, started on an insulin infusion, and then subsequently transferred to our pediatric intensive care unit (PICU). Of note, she had no previous diagnosis of type 1 DM, but it was noted upon further questioning that patient had polydipsia, polyuria, and 10-pound weight loss in the few weeks prior to admission. It was also noted that there was a family history of type 1 and type 2 DM. On exam, she was an obese child with a weight of 90.3 kg and BMI of 35.1 with no acanthosis nigricans noted.

**Table 1:** Patient 1 – Diabetic Ketoacidosis as Evidenced by Initial Laboratory Results

Case 1		
Test	Result	Reference
Blood glucose (mg/dL)	448	65 – 99
Venous blood gas pH	7.08	7.32 – 7.43
Serum bicarbonate (mEq/L)	8	21 – 30
Anion gap (mEq/L)	28	6 – 12
Hemoglobin A1c (%)	11.9	4.8 – 5.6

Initially, the patient was slow to answer questions, tired, and moderately confused. Her Glasgow Coma Scale (GCS) assessment was 14. With treatment, her acidosis improved, and she became more alert. Her acidosis and anion gap resolved, and she was transitioned to subcutaneous rapid-acting insulin (lispro) and long-acting insulin (glargine) on the second day of hospitalization. Soon after transition, she began vomiting and was found to have reverted into DKA with a blood glucose of 240 mg/dL and was put back on an insulin drip. She was switched back to subcutaneous insulin at the end of the third day. Despite being on high potassium-containing fluids, she also had persistent hypokalemia throughout her stay, which took three days to correct.

Her elevated hemoglobin A1c indicated undiagnosed DM for an extended period of time. The patient was discharged home on the fourth day of admission upon established outpatient care with endocrinology. At follow-up, she is doing well with appropriate fasting blood glucose levels and HbA1c of 8.4%. Her anti-glutamic acid decarboxylase (anti-GAD-65) antibodies, htTransglutaminase immunoglobulin A (IgA) antibodies, insulin antibodies, and islet cell antibodies were negative. The patient continues to follow up with Endocrinology.

Case 2

A previously healthy, 17-year-old female presents with altered mental status. On arrival, her initial laboratory testing was consistent with DKA (Table 2). Head imaging and toxicology screens were both negative. Her urinalysis was 1+ positive for ketones. Based on the presentation and labs, it was determined that the patient was presenting in new-onset DKA. She was started on fluids, insulin and transferred to the PICU for further care. Of note, the patient was found to be SARS-CoV-2 positive 10 days before presentation and had been in quarantine when symptoms began. On arrival, she tested positive for SARS-CoV-2 via PCR and IgG antibodies.

**Table 2:** Patient 2 – Diabetic Ketoacidosis as Evidenced by Initial Laboratory Results

Case 2		
Test	Result	Reference
Blood glucose (mg/dL)	2136	65 – 99
Venous blood gas pH	7.07	7.32 – 7.43
Serum bicarbonate (mEq/L)	8	21 – 30
Anion gap (mEq/L)	31	6 – 12
Hemoglobin A1c (%)	14.8	4.8 – 5.6

The patient reported polydipsia and polyuria for weeks prior to presentation and her mother noted unintentional weight loss for the last month. She had no previous diagnosis of DM and no other medical problems. Her elevated HbA1c on admission indicated chronically undiagnosed DM. Based on age, presentation, and normal BMI, the patient was diagnosed with likely new-onset type 1 DM presenting in DKA. Once the anion gap closed and bicarbonate normalized, she was transitioned to subcutaneous rapid-acting insulin (lispro) and long-acting insulin (glargine) on the second day of admission. She was discharged on the fourth day of admission. The patient continues to see Endocrinology.

## Discussion

Here we presented two cases of DKA with new-onset type 1 DM in pediatric patients with concurrent SARS-CoV-2 infection. With the new SARS-CoV-2 pandemic, there has been an increase in DKA in children with a pre-existing diagnosis of type 1 DM [11-12]. The increased incidence may be due to acute infection with SARS-CoV-2 much like other viral illnesses or the result of living in a pandemic where parents are less likely to seek medical care for their children due to fear of exposure or reduced access to primary care services. It has also been noted that there is an increase in the occurrence of new-onset type 1 DM in the pediatric population with few reports of concurrent SARS-CoV-2 infection [5-8].

It is a well-known fact that any viral infection, including SARS-CoV-2, can trigger DKA in a patient with pre-existing DM. Evidence on SARS-CoV-2 triggering new-onset DM in a pediatric patient is very scarce. There are reports of viruses triggering autoimmune type 1 DM in genetically susceptible patients due to massive destruction of pancreatic beta cells [13], so this may be a potential mechanism of SARS-CoV-2 triggering new-onset DM.

The relationship between SARS-CoV-2 infection and the renin-angiotensin-aldosterone system may also play an important role in the pathophysiology of DKA [14]. It is well-described that ACE-2 in the lungs acts as an entry point for the SARS-CoV-2 virus, and the same receptors are also present on the pancreatic cells [15]. The entry of SARS-CoV-2 into pancreatic islet cells may cause direct injury impeding insulin secretion. Second, SARS-CoV-2 antibodies might trigger an inflammatory process leading to autoimmune pancreatic beta-cell destruction and triggering the onset of DM. This second mechanism also explains why some adults have presented with DKA and pancreatitis in the setting of SARS-CoV-2 infection [16-17].

There have been few anecdotal reports worldwide of SARS-CoV-2 infection precipitating DKA in children with no pre-existing diagnosis of type 1 DM [5-8]. However, it is unclear from these reports whether SARS-CoV-2 directly triggered DKA or if concomitant infection precipitated DKA in a child whose diagnosis was delayed due to decreased access to medical services during the pandemic. In both cases, the children had classic symptoms of DM for weeks before presentation, including polydipsia, polyuria, and weight loss. The patients later presented in DKA and were found to be SARS-CoV-2 positive with no symptoms of COVID-19. Both patients had HbA1c levels suggestive of delayed diagnosis. The question remains if the patients’ diagnoses of DM were delayed due to limiting factors in seeking medical attention secondary to the pandemic and if the acute SARS-CoV-2 infection put them into DKA. We wonder if our patients had SARS-CoV-2 infection a few weeks prior to presentation, as that timeline would align with when they began developing symptoms of DM. This would lead us to believe that SARS-CoV-2 may have directly triggered the new-onset DM. We did not obtain SARS-CoV-2 antibodies in the first case, so we are unable to determine if the patient had an acute infection weeks ago and positive PCR may be indicative of prolonged shedding of the virus [18]. SARS-CoV-2 PCR and antibodies were both positive in Case 2. This would support that SARS-CoV-2 may have triggered new-onset DM in an otherwise healthy teenager.

The persistent hypokalemia in our patient, despite the presence of potassium in the intravenous fluids, is also interesting. There have been reports of a higher prevalence of hypokalemia in critically ill patients due to SARS-CoV-2 [19]. This is due to the downregulation of ACE-2 after viral entry, leading to a decreased breakdown of angiotensin II with resultant potassium loss from kidneys from increased secretion of aldosterone [20]. Although hypokalemia is common during the treatment of DKA, the reduction in ACE expression may have also contributed to profound hypokalemia in our case.

There have been many reports on new-onset DM during this pandemic, as well as poor glycemic control in patients with pre-existing DM [11-12]. However, these reports describe type 2 DM with very few incidences of type 1 DM. Our patients were diagnosed with new-onset type 1 DM due to age, family history, and absence of any signs or symptoms of insulin resistance or metabolic syndrome in the setting of SARS-CoV-2 infection.

Our intention with this case report is to raise awareness of a possible link between SARS-CoV-2 and new-onset type 1 DM in children as cases are rarely reported in the US. The increased incidence of DKA may be due to delayed diagnosis secondary to a delay in seeking medical attention during a pandemic or due to SARS-CoV-2 infection itself. Limitations of our case series include that we lack the ability to generalize our findings, it is inferior when compared to prospective analysis, and we are unable to establish causation. Further studies are required to determine a deﬁnitive link and if there is any impact on the severity of DKA in these children with type 1 DM.

## Conclusions

In these two unique pediatric cases, it is plausible that DKA was triggered by SARS-CoV-2 infection, leading to a new-onset type 1 DM. Assuming there is a correlation between these variables, it is unclear if type 1 DM would have ever been diagnosed had the infection not happened. The association of SARS-CoV-2 as a trigger for new-onset diabetes requires further investigation, as the DM incidence has been steadily rising in the pediatric population during the pandemic. Further investigation is necessary to better understand the mechanisms by which SARS-CoV-2 may potentially trigger new-onset DM in children and if so, whether it is a transient or permanent phenomenon.
